# E-cigarette vapor amplifies neutrophilic inflammation and proteolytic EVs in response to LPS

**DOI:** 10.3389/fimmu.2025.1665936

**Published:** 2025-08-27

**Authors:** Lawrence W. Rasmussen, Dakota C. Finley, Julian B. Smith, Aaron S. Noa, J. Edwin Blalock, Amit Gaggar, Matthew C. Madison

**Affiliations:** ^1^ Division of Pulmonary, Allergy and Critical Care Medicine, Department of Medicine, University of Alabama at Birmingham, Birmingham, AL, United States; ^2^ Gregory Fleming James Cystic Fibrosis Research Center, University of Alabama at Birmingham, Birmingham, AL, United States; ^3^ Program in Lung Biology, University of Alabama at Birmingham, Birmingham, AL, United States; ^4^ Lung Health Center, University of Alabama at Birmingham, Birmingham, AL, United States; ^5^ Birmingham VA Medical Center, Birmingham, AL, United States

**Keywords:** neutrophil, elastase, extracellular vesicle, e-cigarette, vaping

## Abstract

**Background:**

While e-cigarette use (vaping) has increased in the last decade, its effects on airway inflammation and extracellular vesicle (EV) biology remain unclear. This study examined how long-term and acute vapor exposures influence lung immune responses, neutrophilic inflammation, and EV-associated proteolytic activity.

**Methods:**

Mice were exposed daily to vapor from commercial e-cigarettes or room air for up to 12 weeks. After exposure, we assessed immune cell recruitment, alveolar damage, and EV populations in the airways. To explore vapor-mediated effects on secondary lung injury, a lipopolysaccharide (LPS) challenge was administered after two weeks of vapor exposure. We then analyzed immune cell responses and isolated neutrophil-derived EVs (nEVs) for transfer into naïve mice to evaluate pathogenic potential.

**Results:**

Vapor exposure alone did not significantly alter immune cell infiltration, lung histology, or EV protease activity. However, mice pre-exposed to vapor and then challenged with LPS showed increased neutrophil infiltration, elevated neutrophil elastase activity in EVs, and greater alveolar damage. Furthermore, nEVs from these mice induced more severe emphysematous changes when transferred to unexposed mice.

**Conclusions:**

While e-cigarette vapor alone does not provoke marked airway inflammation or proteolytic EV release, it creates a primed immune state. This priming amplifies inflammatory and destructive responses to subsequent challenges. These findings suggest vaping may exacerbate lung damage when combined with infections or other environmental stressors, raising concerns about its role in worsening pulmonary disease.

## Introduction

Despite increased regulation worldwide, vaping remains a popular tobacco product, with over 80 million users reported in 2021 (1). The prevalence of adolescents using electronic cigarettes (e-cigarettes) is even more striking, with 4.8% indicating active use in one study (2). The growth in the e-cigarette market stems, in part, from the assumption that the devices are less harmful than traditional cigarettes. Cigarette smoking is a known risk factor associated with the development and progression of pulmonary lung diseases, such as Chronic Obstructive Pulmonary Disease (COPD). Smoking instigates an altered microenvironment in the airway, resulting in delayed mucociliary clearance, mucus hypersecretion, and pronounced airway inflammation (3–5). While *in vitro* and *in vivo* models of e-cigarette exposure have been shown to replicate some physiological characteristics observed in conventional smokers (6–8), whether and how vaping promotes airway inflammation remains poorly understood.

Among inflammatory cells, neutrophils are essential regulators that quickly respond to pulmonary insults, directing a complex armamentarium of functions to promptly remove airway challenges. Neutrophils drive pathogen removal by several mechanisms, including phagocytosis, neutrophil extracellular traps (NETs), and degranulation (9, 10). Recent studies have uncovered an evolving role for neutrophils in generating extracellular vesicles in response to stimuli (11, 12). Neutrophil-released extracellular vesicles (nEVs) can directly influence the airway microenvironment and modulate the physiology of lung cells (13, 14).

Although nEVs can provide protective benefits against airway pathogens (15, 16), persistent pulmonary insults, such as smoking, shift nEVs toward a more harmful proteolytic profile (17). Of particular concern are nEV-specific alterations in the expression of neutrophil elastase (NE), a serine protease responsible for microbial defense and remodeling of the lung’s extracellular matrix (18). At elevated levels, NE has been shown to prompt alveolar damage in COPD/emphysema patients and drive airway obstruction (19). Traditionally, NE’s ability to exceed inhibitory mechanisms, such as local tissue anti-proteases, was attributed solely to soluble forms of NE. However, recent studies demonstrate surface NE on nEVs are a critical source that, in part, overburdens regulatory mechanisms to cause striking parenchymal damage and subsequent loss of pulmonary function (20).

As vaping has been previously shown to disrupt both airway epithelial cell physiology as well as local immune cell function (6, 21–23), there is an urgent need to understand how exposure to e-cigarette vapor might alter lung extracellular matrix biology. However, this sphere of study has been minimally explored to date. Thus, the capacity of vaping to alter nEVs and NE activity represents an unknown risk to pulmonary lung health in a global environment of growing e-cigarette users. The work presented here seeks to provide a model for understanding this dynamic relationship between vaping and NE-bearing nEVs following acute and chronic of lung injury. These insights provide important implications for how exposure to e-cigarette vapor might promote the development of obstructive lung disease or increase the risk of infection and associated complications.

## Materials and methods

### Mice

All murine exposure protocols were reviewed and approved by the University of Alabama at Birmingham Institutional Animal Care and Use Committee (IACUC #20254). 8-week age- and sex-matched C57BL/6 mice were procured from the Jackson Laboratory. Mice were housed in standard cages containing enrichment and bedding in temperature, humidity, and light-controlled rooms with 12-hour light-dark cycles in the animal resource facility of the University of Alabama at Birmingham.

### Vapor exposure

Following a 1-week acclimation period in the UAB Animal Resource Program’s animal facility, mice began the vapor exposure protocol. Mice were exposed to one hour of e-cigarette vapor per day for the designated study period (2 weeks, 6 weeks, or 12 weeks). We implemented a whole-body exposure system (SCIREQ) with the Electronic Nicotine Delivery System (ENDS) extension along with the companion Flexiware 8.0 software. A disposal, draw-activated e-cigarette (Lost Mary (Spearmint) by EBdesign) was implemented to generate vapor. EBdesign manufactures many disposal vapor products popular in the current market. As such, incorporation of Lost Mary enables strong correlations to be drawn from this study towards current e-cigarette users. The device has a 650mAh battery capacity and a nicotine concentration of 40mg/mL with an e-liquid volume of 10mL (4% Nicotine). Nicotine-containing disposable vapor products in the current US market range for 20mg/mL (2% Nicotine) to 50mg/mL (5% Nicotine). Consequently, our 40mg/mL (4% Nicotine) product falls within the range of popular commercial products.

Throughout the one-hour exposure period, the system exposes unrestrained mice to one 3.35-second (55mL) puff of vapor per minute. The 3.35-second puffs of vapor are pumped into the mouse chamber, followed by 56.65 seconds of fresh ambient air before the subsequent puff is initiated. A flowmeter was used to verify pump function and ensure adequate airflow prior to exposing mice to vapor. All tubing and pumps were cleaned thoroughly after each session. No adverse effects of vaping were observed during or post exposure, and zero deaths occurred from the vaping exposure procedure.

### LPS dosing

Following the respective air or vapor exposure periods, mice were anesthetized with isoflurane and treated intratracheally with a single dose of one of the following: Vehicle (50µL sterile saline [0.9%]) or Pseudomonas aeruginosa-derived LPS (Millipore Sigma) (1µg or 35µg) in 50µL sterile saline.

### Immune cell analysis

Bronchoalveolar lavage (BAL) fluid was collected by instilling and removing 0.5 mL of 0.9% clinical-grade saline two times. Saline was pushed manually into mouse lungs through a 20G angiocatheter placed into the trachea. Collected BAL cells were enumerated and spun down onto slides with a cytospin centrifuge and stained using a Hema3 Stain set (23-123869; Fisher Scientific, USA) to calculate differential cell counts of airway macrophages, lymphocytes, and neutrophils.

### Extracellular vesicles harvest and delivery

Mouse EVs were harvested from BAL fluid following the removal of the cellular fraction by using differential ultracentrifugation, as previously described (12, 24). EV size and concentration were determined using the Spectradyne particle analyzer. Afterward, EVs were intratracheally delivered as a single dose of 10^7^ EVs/mouse. This concentration of EVs was previously established as the minimal dose to model the tissue remodeling observed in emphysema (24).

### Histology

Mouse lung tissue was inflated isobarically via the trachea and fixed with 10% buffered formalin for 48 hours. Left lungs were selected for further processing involving paraffin embedding, sectioning, and staining with hematoxylin and eosin. Stained lungs were then imaged (Hamamatsu NanoZoomer S60). Alveolar enlargement was measured blindly by determining the mean linear intercept (L_m_) for each mouse, as previously described (12, 24).

### Neutrophil Elastase assay

Neutrophil Elastase activity was assessed by fluorescence resonance energy transfer (FRET) assays on a SpectraMax id3 plate reader, as previously described (24). In some conditions, EVs were pretreated with NE Inhibitor II (Millipore Sigma) for 30 minutes prior to assessing activity.

### Statistics

Descriptive statistics (mean+/- SEM) were assessed using the Mann Whitney paired t-test. All analyses were set as two-sided with α set to 0.05 to determine significance within GraphPad PRISM Software Inc (ver.9.5.0).

## Results

### Vapor exposure fails to induce neutrophilic inflammation or proteolytic EVs

Despite the lower toxin composition compared to traditional smoke (25, 26), e-cigarette vapor retains several toxic compounds capable of disrupting airway function (6, 21). However, the connection between vaping and airway immunology is less defined. Some studies have indicated distinct immune impairments prompted by vaping (27, 28), while others demonstrate robust inflammatory responses elicited by vapor exposure (7, 29, 30). These disparate observations associated with vapor exposure necessitates a deeper investigation into how e-cigarette use adversely alter the lung’s immune environment.

**Figure 1:**
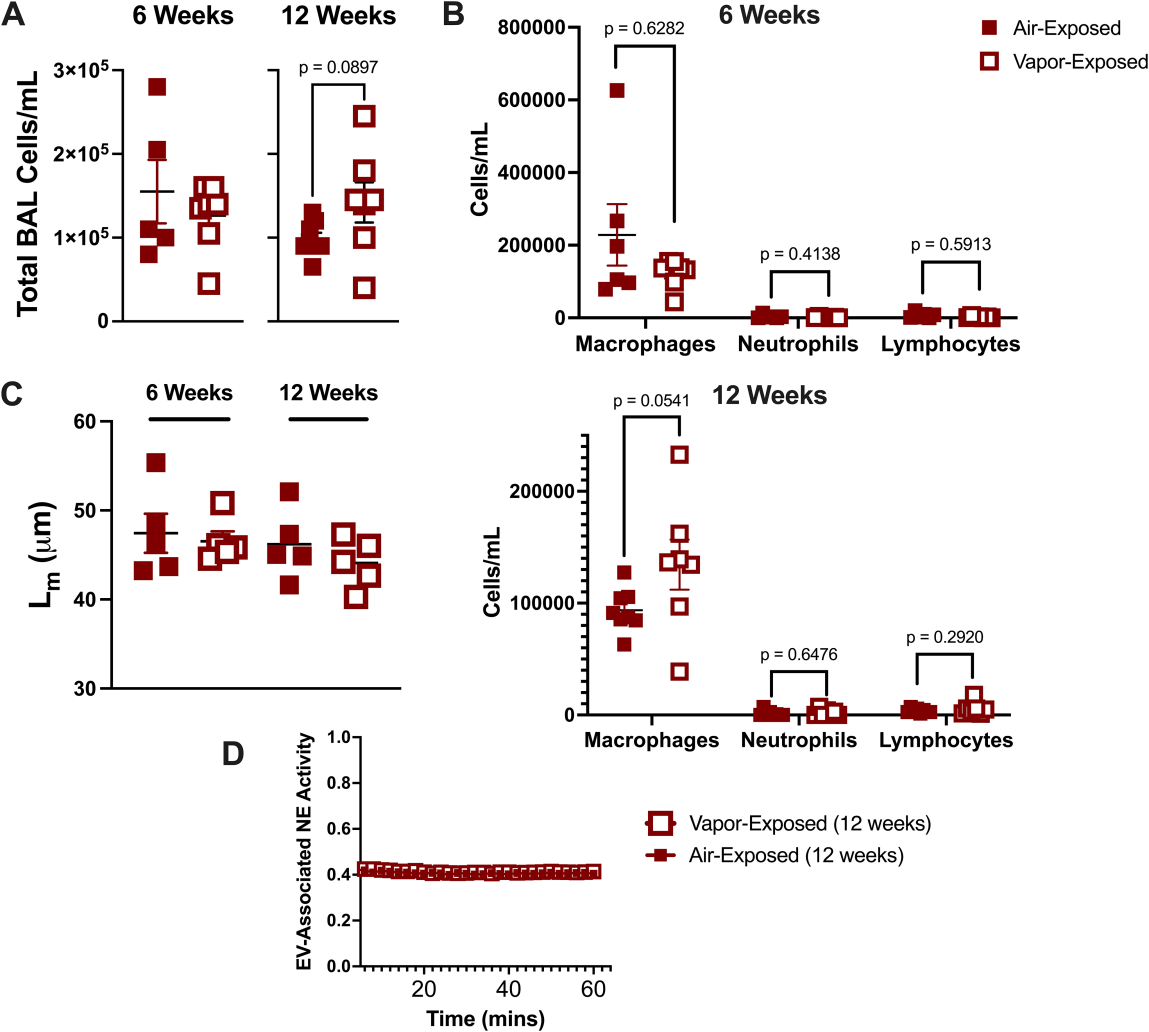
E-cigarette vapor fails to induce inflammation and damage at 6 and 12 week exposures *in vivo*. Following respective exposures in mice to commercial disposable e-cigarettes (6 and 12 weeks), bronchoalveolar lavage (BAL) fluid was harvested. **(A)** BAL Cells were quantified using a hemacytometer. **(B)** Cells were then spun, stained (HEMA 3), and characterized using a cytospin preparation protocol. **(C)** Lung tissue was formalin-fixed, processed, and paraffin embedded. Following H&E staining, mean linear intercept (Lm) was determined for each mouse to score alveolar damage. **(D)** Extracellular vesicles (EVs) were isolated by ultracentrifugation and NE activity was determined by FRET. n≥4 per group. Statistical significance was determined by Mann-Whitney Test.

To better understand how local lung immunity is influenced by vaping, air and vapor exposed mice from 6-week and 12-week exposure cohorts were assessed for changes in airway immune cells. As seen in **Figure 1A**, there was no significant change between the two exposure groups at either the 6- or 12-week time points. Further examination using BAL differential cell counts did not reveal any changes to macrophages, neutrophils, or lymphocytes as a result of vaping duration (**Figure 1B**). Histological assessment of alveolar damage in lung slices, as denoted by L_m_ assessment, revealed no difference in alveolar enlargement between air- and vapor- exposed mice in either the 6- or 12-week cohorts (**Figure 1C**).

Smoke exposure is known to generate a potent, proteolytic EV signature in the airway characterized by enhanced surface expression of proteases, including neutrophil elastase (NE) (20). To examine whether e-cigarette exposure prompted a similar profile of EVs, we isolated airway EV fractions from the 12-week time point and measured NE activity. We observed that in the vapor condition, EVs had no discernable increase NE activity and resembled the EVs derived from the air controls (**Figure 1D**). These data suggest that our model of vapor exposure alone is distinct from traditional smoking and is not sufficient to induce significant neutrophilic inflammation in the airway.

### Acute vapor exposure primes the lung for greater LPS-induced neutrophilic inflammation

**Figure 2:**
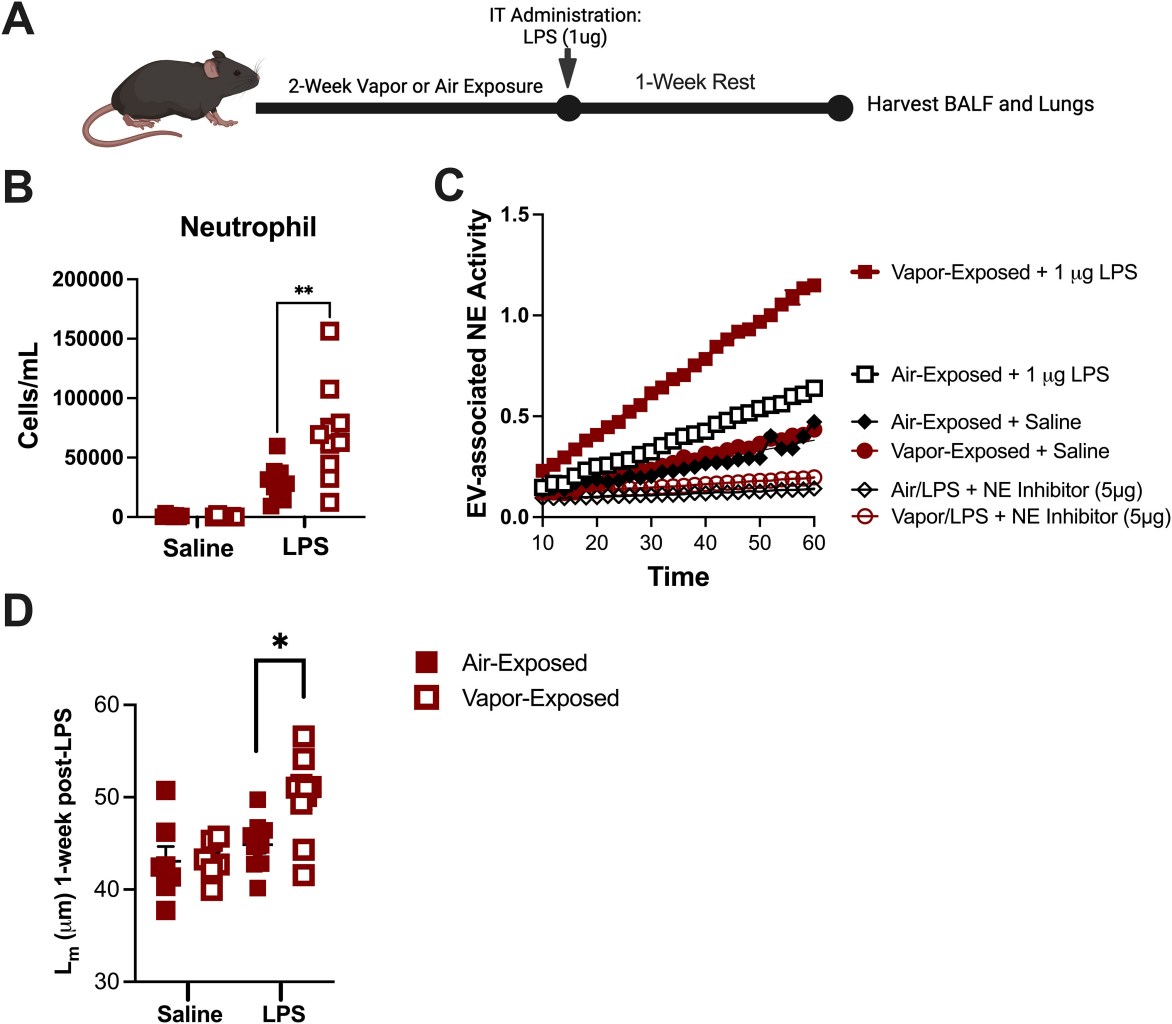
E-cigarette vapor amplifies inflammation and damage associated with LPS *in vivo*. **(A)** Following a 2 week exposure to commercial disposable e-cigarettes, mice were administered LPS (1ug) and allowed to rest with no additional exposures for one week. **(B)** BALF cells were spun and stained (HEMA 3) to allow neutrophils to be quantified using a cytospin preparation protocol. **(C)** Lung tissue was formalin-fixed, processed, and paraffin embedded. Following H&E staining, mean linear intercept (L_m_) was determined for each mouse to score alveolar damage. **(D)** Extracellular vesicles (EVs) were isolated by ultracentrifugation and NE activity was determined by FRET. In some conditions, EVs were pretreated with NE Inhibitor II (5µg). n≥7 per group. Statistical significance was determined by Mann-Whitney Test. *p<0.05, **p<0.01.

Although our model of vapor exposure alone did not induce inflammation in the airway, accumulating evidence suggests that vaping may prime the lung’s immune system. This priming may increase the risk of unintended damage through unknown inflammatory mechanisms in response to a secondary insult, such as microbial infection (28). To evaluate whether vaping caused a priming effect in airway immune cells, mice were first exposed to either air or vapor for 2 weeks. Afterwards, the mice were treated with a one-time vehicle (saline) or LPS dose (1µg) as shown in **Figure 2A**. After dosing, mice were left to recover for one week before evaluating airway inflammation and parenchymal damage. As seen in **Figure 2B**, vapor-exposed mice challenged with 1µg LPS had a significantly higher percentage of neutrophils in the lungs as compared to their air-exposed counterparts (Mean: 16.32% vs. 9.414%). Given the elevated concentration of neutrophils in the airways of vapor-exposed mice, we also sought to evaluate the corresponding nEVs in terms of NE activity. NE activity assays performed with the airway nEVs demonstrated that vapor-exposed mice bore more NE enzymatic activity than the air-exposed counterparts (**Figure 2C**). This increase in activity was NE-specific as pre-treatment with an irreversible NE inhibitor blocked the activity of both Air and Vapor-associated EVs in the assay. Concomitant with the elevated neutrophils and enhanced NE activity, histological analyses revealed that mice also suffered from greater alveolar damage, as indicated increase in L_m_, when mice received prior exposure to e-cigarette vapor (**Figure 2D**). Collectively, these data suggest that although vaping is not capable of inducing neutrophilic inflammation and a proteolytic EV signature on its own, inhaled vapor amplifies inflammatory events prompted by other insults in the lung.

**Figure 3:**
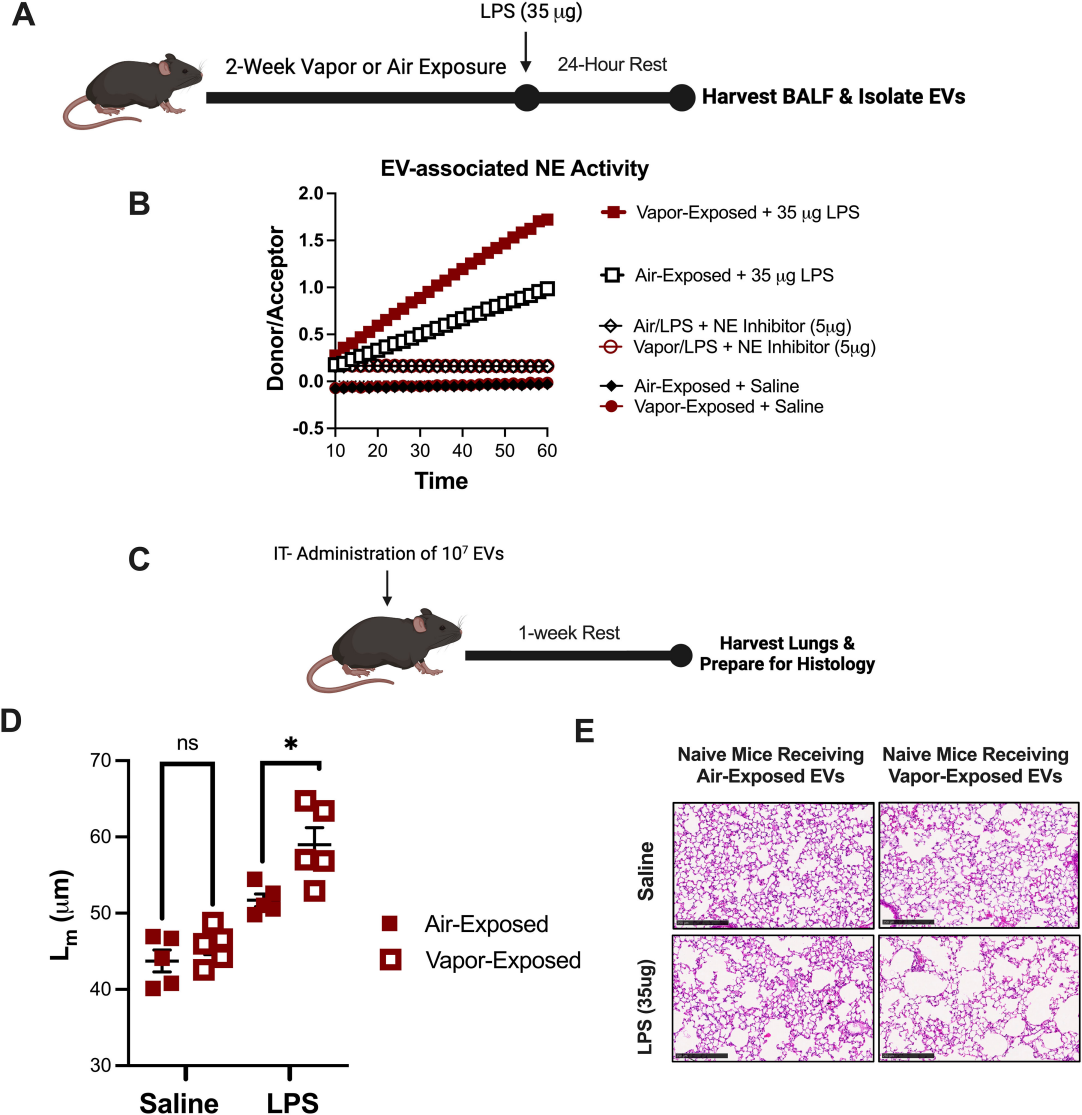
E-cigarette vapor augments an *in vivo* model of EV-induced emphysema. **(A)** Following a 2 week exposure to commercial disposable e-cigarettes, mice were administered LPS (35ug) and airway EVs were harvested 24 hours later. **(B)** Extracellular vesicles (EVs) were isolated by ultracentrifugation and NE activity was determined by a FRET. In some conditions, EVs were pretreated with NE Inhibitor II (5µg). **(C)** 10^7^ EVs (from **B**) were administered to naïve mice intratracheally. Mice were allowed to rest for one week with no additional exposures. Afterwards, lung tissue was harvested, formalin-fixed, processed, and paraffin embedded. **(D, E)** Following H&E staining, mean linear intercept (L_m_) was determined for each mouse to score alveolar damage. Scale bar =100μm. n≥5 per group. Statistical significance was determined by Mann-Whitney Test. *p<0.05. ns, Not Significant.

Our laboratory has previously designed a model of LPS activated, EV-induced emphysema. This model is characterized by marked airway neutrophilia and a heavy burden of NE expressing nEVs in the airway (24). To evaluate whether vapor exposure alters this model and assess whether vaping drives greater inflammatory damage at pathogenic doses of LPS, mice were exposed to either room air or vapor for 2 weeks prior to a one-time dose of either vehicle (saline) or a higher concentration of LPS (35µg) intratracheally (**Figure 3A**). After 24 hours, nEVs from BALF were collected and analyzed from each group to assess changes in nEV expressing NE activity. Confirming our prior observations using the lower dose of LPS (1µg), vaping caused a striking increase in NE activity compared to air-exposed controls (**Figure 3B**). Moreover, increases were NE-specific as pre-treatment with NE inhibitor II blocked the EV-associated NE activity.

To determine the pathogenicity of nEVs in the model, nEVs were instilled intratracheally into naïve recipient mice (10^7^ EVs/mouse) (**Figure 3C**). After one week, mice that received nEVs from vapor-exposed mice challenged with LPS (35µg) demonstrated significantly worsened alveolar enlargement, as indicated by elevated L_m_ measurements (**Figure 3D**). Representative histological images from the mice further illustrate that NE-armed EVs from Vapor+LPS mice prompted greater parenchymal damage and striking emphysema as compared to mice receiving EVs from Air+LPS challenged mice (**Figure 3E**). These findings demonstrate that vaping amplifies the pathogenic potential of LPS-induced nEVs, leading to heightened elastase activity and more severe emphysematous lung damage.

## Discussion

Decades of research on traditional tobacco smoke have shaped our understanding of lung disease and supported the development of robust model systems, including those that have illuminated EV biology (12, 20, 25). These models are also supported by decades of extensive clinical and epidemiological data, enabling translational insights between human and animal studies. In contrast, e-cigarettes have only been in commercial use since the early 2000s and lack comparable modeling frameworks. Although the products remain extremely popular among adolescent and adult populations, the research into how exposure to e-cigarettes impact airway EVs and host defense mechanisms is still very limited. Thus, development of translational and scalable models is imperative to deepen our understanding of the biological consequences of vaping and mitigate associated disease risks.

Here, we present a murine model enabling investigation into the influence of e-cigarette vapor on pulmonary neutrophil physiology and the airway EV environment. Unlike traditional cigarette smoke, vapor exposure alone does not vigorously recruit neutrophils. However, our data suggests vaping does prime the lung for exaggerated neutrophil-mediated immune responses to secondary insults. Although vapor-exposed mice showed minimal inflammation under baseline conditions, the mice displayed heightened neutrophilic inflammation and alveolar damage following LPS challenge, effects that persisted up to one week post-LPS challenge. These harmful responses within the hosts’ lungs reflect findings in previously reported infection models, where vaping worsened outcomes following microbial challenge. Exposure to e-cigarette vapor has been shown to increase biofilm formation on airway cells (31), enhance the virulence of *Staphylococcus aureus* in pneumonia models (31), and raise *Pseudomonas* burden in sepsis models (22). Likewise, viral infection models using vapor-exposed mice demonstrate a similar phenomenon. Vaping did not induce adverse lung inflammation in mice, but was shown to induce worsening outcomes when followed by influenza A infection, namely higher mortality and prolonged lung damage (28). Overall, these findings strongly suggest e-cigarette vapor may appear benign under baseline conditions, but it harbors the capacity of priming the respiratory system for exaggerated injury and prolonged inflammation in the face of secondary infectious challenges.

Furthermore, our data aligns with human studies demonstrating that vaping alters neutrophil phenotype and protease activity (9, 30, 32). Increased NE and matrix metalloproteinase-9 (MMP-9) activity has been previously observed in the airways of vaping subjects and is correlated with dysregulated proteolytic activity (9). Our data extends upon these findings by demonstrating high expression of NE on the surface of airway EVs. This is of strong clinical significance as EV-associated NE resists inhibition by native anti-proteases, contributing to persistent extracellular matrix degradation and promoting a cycle of exaggerated neutrophil-mediated inflammation in the lung (12). In our model, vapor + LPS, even at low doses (1µg), significantly elevated EV-associated NE activity, implicating vaping’s role in sustained tissue injury after minor insults. This has strong translational relevance because, unlike laboratory mice which are often housed in a germ-free facility, human lungs are continuously exposed to environmental insults such as pollutants, allergens, and pathogens. This wide array of daily inhaled challenges shapes the local pulmonary immune environment by altering the function and composition of resident and recruited immune cells. To the best of our knowledge, the data presented here is the first to demonstrate that vapor-generated nEVs are an underappreciated but important regulator of lung disease. Our current findings expand the nEV paradigm of lung injury and strongly suggest that these bioactive particles should be considered as both mechanistic drivers and possible prognostic indicators to susceptible populations exposed to environmental exposures.

Collectively, our work underscores a key mechanistic insight for e-cigarette users: vaping primes neutrophil responses and EV-mediated proteolysis in a way that may not manifest without a secondary insult. This novel observation supports a “second-hit” model of lung pathology, with important implications for susceptible individuals who may be more vulnerable to infection or environmental exposures. Additionally, as tobacco use evolves to incorporate more polytobacco users (33), these data strongly suggest more systemic investigations into dual use (concurrent vaping and smoking). Vaping’s capacity to induce a harmful profile through nEVs may exacerbate the pre-existing smoke-induced neutrophilic inflammation, resulting in more extreme and faster progression of pulmonary lung diseases, such as emphysema.

Although these observations are novel and provide important physiological implications to e-cigarette use, a notable limitation to this model is the use of a single commercial vaping product, Lost Mary (Spearmint). There are numerous brands of e-cigarettes encompassing a myriad of flavors, which may influence the severity observed in our second-hit model. Lost Mary (Spearmint) was selected as a representative e-cigarette, containing 4% nicotine, which is in the range of similar products, like JUUL (34). However, given the strong results from our second-hit model, we are now engaging in ongoing studies to examine the impact of other common brands of e-cigarettes as well as different nicotine and flavor compositions on neutrophil-associated EV signatures. These studies will also broaden inspection of variables such as age and sex, to better represent the range of the vaping community. Building upon the core strength of this model, the accelerated timeline (2–3 weeks) enables expeditious identification of the potential risks faced by e-cigarette users. Furthermore, our model’s rapid, cost efficient, and adaptable framework offers a valuable tool for expanding these observations into these new investigative areas. Importantly, our incorporation of nEV analysis also opens promising avenues for therapeutic intervention, particularly in targeting aberrant neutrophil-driven inflammation.

## Data Availability

The raw data supporting the conclusions of this article will be made available by the authors, without undue reservation.
